# Pronostic des références obstétricales à l’Hôpital Gynéco-Obstétrique et Pédiatrique de Yaoundé (HGOPY)

**DOI:** 10.11604/pamj.2017.28.301.10773

**Published:** 2017-12-08

**Authors:** Etienne Belinga, Pascal Foumane, Same Julius Dohbit, Esther Meka Ngo Um, Daniel Kesseng Kiyeck, Emile Télesphore Mboudou

**Affiliations:** 1 Département de Gynécologie-Obstétrique, Faculté de Médecine et des Sciences Biomédicales Université de Yaoundé I, Yaoundé, Cameroun

**Keywords:** Pronostic, référence obstétricale, complications maternelles, décès périnatal, Cameroun, Prognosis, referred patients with an obstetric emergency, maternal complications, perinatal deaths, Cameroon

## Abstract

**Introduction:**

L’impact des références sur la survenue des complications obstétricales n’est pas encore connu. Notre but était d’identifier les complications associées aux références obstétricales à Yaoundé.

**Méthodes:**

Il s’agissait d’une étude transversale descriptive et analytique du 1^er^ Février au 31 Juillet 2015 à l’Hôpital Gynéco-Obstétrique et Pédiatrique de Yaoundé, ayant comparé les femmes référées aux non référées et leurs nouveau-nés respectifs. L’échantillonnage était consécutif et exhaustif pour l’analyse des caractéristiques sociodémographiques, obstétricales et néonatales précoces. Les tests de Chi carré et exact de Fisher ont aidé à comparer les variables qualitatives. L’analyse par régression logistique a permis d’éliminer les facteurs confondants. P était significatif si p < 0,05.

**Résultats:**

Après analyse multi variée, les complications obstétricales statistiquement significatives chez les patientes référées étaient: la rupture prématurée des membranes (OR ajusté = 9,37, IC95%: 2,52-66,98, p = 0,002); la prématurité (OR ajusté = 4,14 (1,88-9,16; P < 0,001) et le décès après asphyxie néonatale sévère (OR ajusté = 6,48 (1,17-35,80); P = 0,032).

**Conclusion:**

La rupture prématurée des membranes, la prématurité et le décès après asphyxie néonatale sévère sont les complications associées aux références obstétricales à Yaoundé.

## Abstract (en)

**Introduction:**

The impact of referred patients with an obstetric emergency due to complications is not yet known. This study aimed to identify the complications associated with obstetric emergency in referred patients in Yaoundé.

**Methods:**

We conducted a cross-sectional descriptive and analytical study at the Yaoundé Gynaecology, Obstetrics and Pediatrics Hospital from 1 February to 31 July 2015. We compared the women referred to the Emergency Department with the non-referred women and their newborns respectively. Consecutive and comprehensive sampling technique was used for early analysis of the sociodemographic, obstetric and neonatal features. Chi-squared tests and Fisher exact probability test helped to compare the qualitative variables. Logistic regression analysis helped to eliminate the confounding factors. The significant level was set at p < 0.05.

**Results:**

After multivariate analysis, statistically significant obstetric complications in referred patients were: premature rupture of membranes (adjusted OR = 9.37, CI 95%: 2,52-66,98, p = 0.002); preterm birth (adjusted OR = 4.14 (1,88-9,16; p < 0.001) and death after severe neonatal asphyxia (adjusted OR = 6.48 (1,17-35,80); p = 0.032).

**Conclusion:**

Premature rupture of the membranes, preterm birth and death after severe neonatal asphyxia are the complications associated with obstetric emergency in referred patients in Yaoundé.

## Introduction

Les références obstétricales sont associées à 70-90% des décès maternels enregistrés dans les hôpitaux selon Perrin et al. 2012 [1]. Bien que ces références visent à réduire la morbi-mortalité materno-fœtale, les complications spécifiques aux patientes référées sont peu documentées dans notre environnement. Le but de cette étude était de contribuer à l’identification de celles-ci à l’Hôpital Gynéco-Obstétrique et Pédiatrique de Yaoundé (HOGPY) au Cameroun.

## Méthodes

**Type d’étude**: Il s’agissait d’une étude transversale descriptive et analytique.

**Période de l’étude**: Du 1^er^ Février au 31 Juillet 2015.

**Lieu de l’étude**: La maternité de l’Hôpital Gynéco-Obstétrique et Pédiatrique de Yaoundé.

**Population d’étude**: Nous avons comparé deux groupes de femmes en travail ou en post-partum précoce. Le premier groupe était constitué de femmes référées (groupe 1) et le second groupe constitué de femmes non référées (groupe 2) ainsi que leurs nouveau-nés respectifs.

**Critères d’inclusion**: Nous avons inclus toutes les femmes admises en salle de travail avec une grossesse d’au moins 28 semaines d’aménorrhée ou en période post-partum précoce ayant consenti à participer à l’étude.

**Critères de non inclusion**: Les césariennes électives, les femmes non enceintes ou les femmes enceintes dont l’âge gestationnel était inférieur à 28 semaines d’aménorrhée n’ont pas été incluses.

**Taille de l’échantillon**: La formule proposée par Shulz et al en 2005 [2] a permis de calculer une taille minimale de l'échantillon de 108 sujets par groupe sur la base d'un taux de césarienne de 19,7% rapporté par Foumane et al. 2014 sur le site de l'étude [3], en supposant que le risque de césarienne serait multiplié par 2 chez les patientes référées.

**Echantillonnage**: Il était consécutif et exhaustif.

**Considérations éthiques**: Avant de mener cette étude, nous avons au préalable obtenu une autorisation de recherche du Comité Institutionnel d’Ethique de la Recherche pour la Santé Humaine (CIERSH) de l’Hôpital Gynéco-Obstétrique et Pédiatrique de Yaoundé et dans le respect de la personne humaine nous avons remis à chaque participante une fiche d’information et de consentement avec explications au besoin. Les données recueillies ont été gardées confidentielles et anonymes pour toutes les participantes.

**Collecte des données**: Les données ont été recueillies à l’aide d’un formulaire standardisé et pré-testé auprès des patientes ayant consenti à participer à l’étude. Les variables recueillies chez les parturientes étaient: les données sociodémographiques, les antécédents obstétricaux et personnels, la structure de référence, le délai de référence, le motif d’admission, l’histoire de la grossesse actuelle, les données de l’examen clinique, le diagnostic, la prise en charge, le déroulement du travail, le mode d’accouchement, la survenue ou non d’une hémorragie du post partum, la fièvre maternelle et le décès maternel. Chez le nouveau-né nous avons recueilli le terme de naissance, le poids de naissance, le score d’Apgar à la première et la cinquième minute, le transfert ou non en néonatalogie, l’asphyxie néonatale et le décès après asphyxie néonatale sévère ou non.

**L’analyse des données**: Elle s’est faite avec le logiciel Epi info 3.5.4 et SPSS 14.0. Le test de Chi carré et le test exact de Fisher ont aidé à comparer les variables qualitatives. Le Rapport de côte (OR) et le Risque Relatif (RR) avec leurs intervalles de confiance à 95% ont été calculés pour évaluer l’association entre les variables. Une analyse par régression logistique pour éliminer les facteurs de confusion a été effectuée. La valeur de p était significative lorsque p était < 0,05.

## Résultats

Au total nous avons recruté 323 femmes dont 121 femmes référées (37,46%) et 202 femmes non référées et un total de 351 nouveau-nés. La tranche d’âge la plus représentée était 20-29 ans, aussi bien chez les femmes référées (55,4%) que chez les non référées (48,5%). Après analyse univariée, nous avons observé (Tableau 1) que les parturientes référées étaient 16,15 fois non scolarisées plus que les non référées (P = 0,001). Le niveau d’étude était surtout primaire (OR=2,13 (IC 95%: 1,19-3.81) p = 0,007). Elles étaient 3,57 fois plus susceptibles de n’avoir pas fait d’échographie (P = 0,001) et 2,04 fois susceptibles d’avoir pris moins de 3 doses de traitement préventif intermittent du paludisme (TPI) (P = 0,001). Ces femmes référées avaient 5,15 fois plus de chances d’avoir fait moins de 4 consultations prénatales (CPN) (P < 0,001) et étaient très souvent suivies dans un centre de santé (OR = 5,96 (IC 95%: 3,48-10,24); P < 0,001). Sur le plan clinique une hauteur utérine < 29cm était très fréquente chez les femmes référées (OR = 6,19 (IC95%: 2.56-14.99); P < 0,001) ainsi que la rupture prématurée des membranes (OR=2,06 (IC95%: 1,52-2,79); p < 0,001) (Tableau 2). Les complications significatives chez les parturientes référées en analyse univariée étaient les suivantes: la rupture des membranes (RR = 3,18 (IC: 1,99-5,09); p < 0,001), la pré éclampsie sévère (RR = 1,70 (IC: 1,19-2,44); p < 0,015), la menace d’accouchement prématuré (RR = 1,66 (IC: 1,22-2,26); p < 0,004), l’infection santé partum (RR=2,27 (IC: 1,55-3,35); p < 0,029), le syndrome de pré rupture (RR = 2,72 (IC: 2,36-3,15); p = 0,019), le poids de naissance < 2500g (RR = 1,81 (IC: 1,41-2,34); p < 0,001), le score d’Apgar à la 5^ème^minute < 7 (RR = 1,67(IC:1,26-2,24); p = 0,002), le transfert en néonatalogie (RR = 1,50 (IC:1,15-1,94); p = 0,002), la souffrance fœtale aigüe (RR=1,38 IC:1,04-1,83); p = 0,023), la prématurité (RR = 1,80 (IC: 1,40-2,32); p < 0,001), l’asphyxie néonatale (RR=1,85 (IC: 1,40-2,45); p < 0,001), le décès périnatal (RR = 1,59 (IC:1,19-2,13); p = 0,004) et enfin le décès après asphyxie néonatal sévère (RR = 1,69 (IC: 1,05-2,70); p = 0,0035) (Tableau 3). Après analyse multi variée par régression logistique, les seules facteurs retenus comme significativement associés aux complications des références obstétricales étaient: la rupture prématurée des membranes (OR ajusté = 9,37, (IC 95%: 2,52-66,98); p = 0,002); la prématurité (OR ajusté = 4,14 (IC 95%: 1,88-9,16); p < 0,001) et le décès par asphyxie néonatale sévère (OR ajusté = 6,48 (IC 95%: 1,17-35,80); p = 0,032).

**Tableau 1:** Variables maternelles et fœtales associées à la référence obstétricale (analyse univariée)

VARIABLES	^+^Groupe 1 N (%)	^++^Groupe 2 N (%)	OR (IC à 95%)	Valeur de P
non scolarisée	9 (7,4)	10,50	16,15 (2,02-129,14)	0,001
Niveau d’instruction primaire	30 (24,8)	27 (13,4)	2,13 (1,19-3,81)	0,007
Ménagère	42 (34,7)	43 (21,3)	1,96 (1,19-3,25)	0,006
Employée du secteur informel	30 (24,8)	29 (14,4)	1,96 (1,11-3,47)	0,014
Sans revenu fixe	30(24,8)	30 (14,9)	1,88 (1,06-3,33)	0,019
CPN < 3	51(42,1)	25 (12,4)	5,15 (2,97-8,97)	< 0,001
Aucune échographie réalisée	19(15,7)	10(5)	3,57 (1,60-7,98)	0,001
Échographies réalisées 1 à 2	64(52,9)	83 (41,1)	1,60 (1,02-2,53)	0,025
TPI 1 à 2 doses	78(64,5)	95 (47)	2,04 (1,29-3,25)	0,001
CPN à l’Hôpital de District	25(20,7)	14 (6,9)	3,49 (1,73-7,03)	< 0,001
CPN dans un Centre de Santé	58(47,9)	27 (13,4)	5,96 (3,48-10,24)	< 0,001
CPN suivies par un Infirmier	40(33,1)	17 (8,4)	5,37 (2,88-10,03)	< 0,001
CPN par un Infirmier accoucheur	28(23,1)	12 (5,9)	4,76 (2,31-9,79)	<0,001
Perte des eaux	42(34,7)	32 (15,8)	2,82 (1,66-4,80)	< 0,001
HU < 29 cm	22(18,2)	7 (3,5)	6,19 (2,56-14,99)	< 0,001
CA entre] 60-90] cm	33(27,3)	26(12,9)	2,53 (1,42-4,50)	0,001

+ **Groupe 1:** Référées

++ **Groupe 2:** Non référées** CPN**: consultation prénatale; **TPI**: traitement préventif intermittent contre le paludisme; **HU**: hauteur utérine; **CA**: circonférence abdominale

**Tableau 2:** Complications maternelles et fœtales associées à la référence obstétricale (analyse univariée)

VARIABLES	Groupe 1^+^ N (%)	Groupe 2^++^ N (%)	RR (IC à 95%)	Valeur de P
Membranes Rompues	79 (65,3)	75 (37,1)	3,18 (1,99-5,09)	< 0,001
Pré éclampsie sévère	14 (11,6)	9 (4,5)	1,70 (1,19-2,44)	0,015
Menace d’accouchement prématuré	23 (19)	17 (8,4)	1,66 (1,22-2,26)	0,004
Infections anté partum	5 (4,1)	1 (0,5)	2,27 (1,55-3,35)	0,029
Syndrome de pré rupture	4 (3,3)	0 (0)	2,72 (2,36-3,15)	0,019
Poids de naissance < 2500 g	54 (40,3)	41 (18,9)	1,81 (1,41-2,34)	<0,001
Apgar à la 5^ème^ minute < 7	26 (19,4)	18 (8,3)	1,67 (1,26-2,24)	0,002
Réanimation néonatale	66 (49,3)	70 (32,3)	1,53 (1,18-1,99)	0,001
Transfert en néonatalogie	62 (46,3)	66 (30,4)	1,50 (1,15-1,94)	0,002
Souffrance fœtale aigüe	37 (27,6)	39 (18)	1,38 (1,04-1,83)	0,023
Prématurité	50 (37,3)	37 (17,1)	1,80 (1,40-2,32)	< 0,001
Asphyxie néonatale	24 (17,9)	13 (6)	1,85 (1,40-2,45)	< 0,001
Décès périnatale	27 (20,1)	21 (9,7)	1,59 (1,19-2,13)	0,004
Décès après asphyxie néonatal sévère	13 (48,1)	4(19)	1,69 (1,05-2,70)	0,035

+ **Groupe 1**: Référées

++ **Groupe 2**: Non reférées

**Tableau 3:** Facteurs significatifs materno-fœtaux associés aux références (analyse multi variée)

VARIABLES	OR ajusté	IC à 95%		Valeur de P
		Inférieur	Supérieur	
CPN ≤ 3	4,32	0,13	0,94	0,037
Nulliparité	8,57	5,84	74,06	0,003
Aucun Revenu	8,25	0,01	0,42	0,004
RPM	9,37	2,52	66,98	0,002
Prématurité	4,14	1,88	9,16	<0,001
Décès après asphyxie néonatale sévère	6,48	1,17	35,80	0,032

RPM: Rupture Prématurée des Membranes

## Discussion

La fréquence des patientes référées dans notre série était de 37,46%. Ce taux est proche de celui retrouvé par Tshabu-Aguèmon et al. 2012 au Benin (30,38%) [4] et Thiam et al. 2013 au Sénégal (31,2%) [5]. Par contre, nos résultats sont supérieurs à ceux de Diarra et al. 1999 en Côte d’Ivoire (7,9%) [6] et Louai et Baskett 2007 en nouvelle Ecosse (1,3%) [7] Les nullipares étaient 8,57 fois plus souvent référées que les non nullipares. Elles représentaient 40,5% des parturientes référées. Nos résultats sont semblables à ceux de Thiam et al. 2013 [5] (35,2%), et de Diarra et al 1999 [5] 27,7%. Par contre, nos résultats sont supérieurs à ceux de la série de Sépoua et al. [8] en Centrafrique 5,8% de nulligestes chez les femmes évacuées. Les études que nous avons consultées étaient toutes des études descriptives et ne permettaient pas de chiffrer le lien entre la nulliparité et les références obstétricales. Les femmes transférées avaient significativement un nombre de consultations prénatales (CPN) inférieur à 4 (OR = 5.15; P < 0,001) (Tableau 1). Ces données sont semblables à celles de Thiam et al. 2013 [5] pour qui deux femmes sur trois avaient moins de 4 CPN. Au-delà du faible nombre, ces CPN étaient de mauvaise qualité dans notre série. En effet, moins de 3 doses de TPI contre le paludisme étaient prises (P = 0,001), le suivi de la grossesse était significativement assuré au centre de santé (p < 0,001) par des personnels peu qualifiés. D’après Foumane et al 2014 et en 2016 au Cameroun, la mauvaise qualité des CPN est un risque d’issue défavorable pour la mère et le nouveau-né [3, 9]. La hauteur utérine inférieure à 29 cm était significativement présente chez les femmes référées (OR = 6,19 P < 0,001) ainsi qu’un âge gestationnel inférieur 37 SA (OR = 3,40; P < 0,001). Ces trouvailles sont en accord avec le fait que la prématurité reste très significativement associée aux références obstétricales aussi bien en analyse univariée qu’en analyse multivariée (OR ajusté 4,14; p < 0,001). La rupture prématurée des membranes(RPM) est apparue significative chez les femmes référées même après analyse multivariée (OR ajusté 4,14; P < 0,001). La série de Louai et Baskett en 2007 a décrit 21% de RPM chez les référées en Nouvelle Ecosse [7]. Par contre, les études africaines parcourues ne la décrivent pas. La RPM est connue pour augmenter le risque infectieux maternel et fœtal, justifiant son association avec la référence obstétricale. Le syndrome de pré rupture est significativement retrouvé chez les parturientes référées (OR = 2,72; p = 0,019) dans ce travail. Cette donnée est similaire à celles rapportées par Diarra et al. en 1999 [6] et Tshabu-Aguèmon et al. en 2012 [4]. Chez les bébés nés des femmes référées le décès après asphyxie néonatale était statistiquement significatif aussi bien en analyse univariée qu’en analyse multivariée (OR ajusté = 6,48; IC 95%: 1,17-35,80; p = 0,032) et l’asphyxie néonatale était augmentée de 1,85 fois (P < 0,001) avec 27 décès néonataux (20,1%) observés. Nos résultats sont en accord avec ceux de Nkwabong et al [10], 26 cas de décès néonataux (21,3%) alors qu’ils diffèrent de ceux de Foumane et al [9] (0.93%), Sandjong et al (1,8%) [11]. La différence est due à notre population cible qui était constituée de femmes référées.

## Conclusion

La rupture prématurée des membranes, la prématurité et le décès après asphyxie néonatale sévère sont des complications qui affectent significativement de façon indépendante les femmes référées et leurs nouveau-nés.

### Etat des connaissances actuelles sur le sujet

- La référence obstétricale vise à réduire la morbi-mortalité materno-fœtale;
- Les références obstétricales sont associées à 70-90% des décès maternels enregistrés dans les hôpitaux;
- Les données spécifiques de l’Hôpital Gynéco-Obstétrique et pédiatrique de Yaoundé ne sont pas connues.

### Contribution de notre étude à la connaissance

- Les complications spécifiques auxquelles sont exposées les femmes référées à l’Hôpital Gynéco-Obstétrique et pédiatrique de Yaoundé et leur nouveau-nés sont les suivantes: la rupture prématurée des membranes; la prématurité; le décès après asphyxie néonatale sévère.

## Conflits d’intérêts

Les auteurs ne déclarent aucun conflit d'intérêts.

## Remerciements

Les auteurs remercient le personnel de la salle d’accouchement et d’hospitalisation de la maternité de l’Hôpital Gynéco-obstétrique et Pédiatrique de Yaoundé pour leur collaboration tout au long de cette étude.
